# Echocardiographic Assessment of Patients Undergoing Mitral Valve Repair

**DOI:** 10.3390/jcdd12120498

**Published:** 2025-12-17

**Authors:** Marco Rolando, Nadeem Elmasry, Federico Gobbi, Antonella Moreo, Nina Ajmone Marsan, Erberto Carluccio, Federico Fortuni

**Affiliations:** 1 Independent Researcher, 63100 Ascoli Piceno, Italy; 2 Department of Cardiology, Leiden University Medical Centre (LUMC), Albinusdreef 2, 2300 RC Leiden, The Netherlands; 3 Cardiology and Cardiovascular Pathophysiology, S. Maria Della Misericordia Hospital, University of Perugia, Piazzale Menghini 1, 06129 Perugia, Italy; 4 Department of Cardiology, De Gasperis Cardio Center, ASST Grande Ospedale Metropolitano Niguarda, Piazza dell’Ospedale Maggiore 3, 20162 Milan, Italy

**Keywords:** mitral regurgitation, three-dimensional echocardiography, multimodality imaging, multidisciplinary approach, transcatheter mitral valve intervention, transcatheter edge-to-edge repair

## Abstract

Mitral regurgitation (MR) is one of the most prevalent valvular disorders worldwide, with a growing burden driven by population aging and improved diagnostic capabilities. Understanding the mechanism of MR, whether primary, due to intrinsic valve abnormalities, or secondary, resulting from atrial or ventricular remodeling, is essential for optimal management. Echocardiography, particularly advanced modalities such as three-dimensional imaging and strain analysis, plays a central role in this process. It allows accurate quantification of MR severity, detailed characterization of valve and ventricular anatomy, and assessment of remodeling, all of which are critical for determining the optimal timing for intervention. Beyond diagnosis, echocardiography is indispensable in guiding therapy selection: it informs surgical planning by defining leaflet pathology for repair versus replacement strategies, and directs transcatheter interventions by guiding interatrial septal puncture, catheter orientation, and device deployment in real time. While surgery remains the gold standard for primary MR, transcatheter approaches including edge-to-edge repair and emerging mitral valve replacement are increasingly relevant, particularly in patients at high surgical risk or with complex anatomy. This review emphasizes the pivotal role of echocardiography in the pre-procedural assessment of MR, highlighting its ability to integrate anatomical, functional, and hemodynamic information to guide patient-tailored therapeutic strategies and optimize outcomes within a Heart Team framework.

## 1. Introduction

Mitral valve disease (MVD) is one of the most prevalent valvular diseases worldwide, with specific etiologies and prognostic implications. Mitral regurgitation (MR) can arise from congenital or acquired abnormalities of the MV. Among acquired causes, the two major forms are degenerative MR (DMR) and functional MR (FMR), which represent the most common mechanisms encountered in adult patients and form the focus of this review. It is also important to recognize that MR may present with a mixed etiology, in which both degenerative and functional components contribute to the severity of regurgitation. DMR arises from structural abnormalities of the valve itself, such as leaflet degeneration, chordal rupture, or rheumatic damage, meaning the lesion is intrinsic to the valvular apparatus. FMR, on the other hand, occurs when the valve is structurally normal but fails to close properly because of changes in the geometry and function of the left ventricle (LV) or left atrium (LA). Within FMR, two entities can be further distinguished:

- Ventricular FMR (v-FMR): it is typically associated with ischemic or non-ischemic cardiomyopathy, where adverse LV remodeling leads to papillary muscle displacement, leaflet tethering, and annular dilatation [1].
- Atrial FMR (a-FMR): increasingly recognized as a distinct condition, it is driven by LA dilatation, atrial myopathy, and atrial fibrillation (AF), usually in the presence of preserved LV systolic function [2].

These two phenotypes not only differ in pathophysiology but also in prognosis and potential response to therapy [3,4]. The distinction has important clinical implications, as they require tailored diagnostic and therapeutic strategies.

In high-income countries, DMR predominantly arises from fibroelastic deficiency and Barlow’s disease, whereas rheumatic heart disease remains the leading cause in low- and middle-income countries. Accurate estimation of FMR prevalence is hampered by heterogeneity in patient cohorts and coexistence of ischemic and non-ischemic disease. DMR is also more common in young patients, whereas FMR is predominantly encountered in older individuals [5,6].

**Figure 1:**
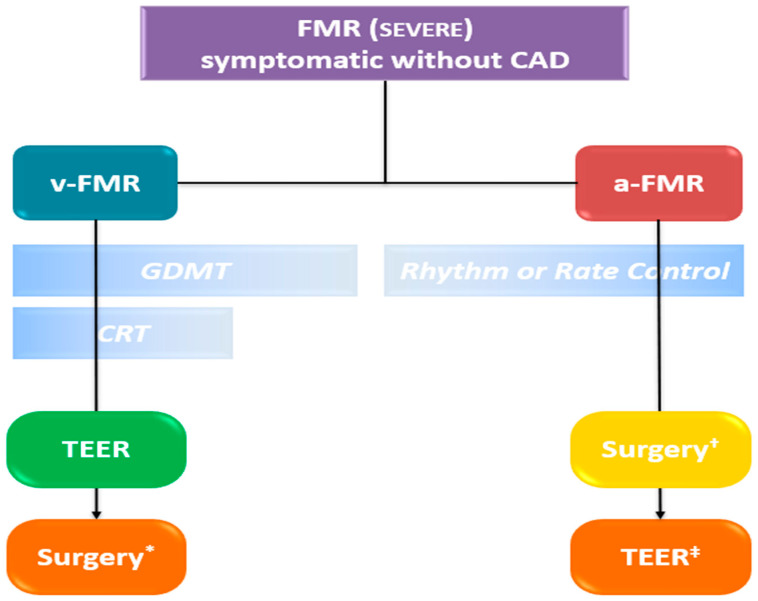
Treatment algorithm for severe FMR in patients without associated coronary artery disease. * If anatomical features preclude TEER feasibility. ^†^ In patients with acceptable surgical risk. ^‡^ In patients with high surgical risk. a-FMR: atrial functional mitral regurgitation; CAD: coronary artery disease; CRT: cardiac resynchronization therapy; FMR: functional mitral regurgitation; GDMT: guideline-directed medical therapy; TEER: transcatheter edge-to-edge repair; v-FMR: ventricular functional mitral regurgitation.

For decades, surgery has been the gold standard in the management of DMR, with repair systematically outperforming replacement, providing excellent durability and long-term outcomes in experienced centers, in contrast to FMR where surgical interventions have yielded less consistent benefits [7,8]. Perioperative risk may represent a substantial limitation in elderly and comorbid patients; however, minimally invasive surgical approaches have significantly reduced operative trauma, still preserving outstanding efficacy [9]. Over the past decade, transcatheter therapies have profoundly reshaped treatment strategies. Although transcatheter edge-to-edge repair (TEER) was first tested in DMR cohorts, its most transformative role has emerged in FMR [10,11,12]. In this setting, TEER redefined the treatment paradigm by providing a less invasive yet clinically effective option, with surgery largely limited to patients whose anatomy precludes TEER (Figure 1) [13].

Meanwhile, transcatheter mitral valve replacement (TMVR) has further expanded the therapeutic spectrum, offering predictable MR elimination in patients with complex anatomy, failed prior surgical repair, or unsuitable conditions for TEER, although long-term durability and procedural risks such as LV outflow tract (LVOT) obstruction remain areas of ongoing investigation [14].

Together, these advances mark a paradigm shift from a predominantly surgical strategy to a landscape of several potential alternatives—ranging from transcatheter to surgical approaches—that require patient-tailored selection.

The rapid evolution of imaging has been a key driver of this transformation. Conventional bidimensional (2D) echocardiography remains the cornerstone of MR assessment, but three-dimensional (3D) echocardiography, speckle-tracking echocardiography, cardiac magnetic resonance (CMR), and computed tomography (CT) have expanded our ability to characterize anatomy, assess chamber remodeling, and quantify severity with greater accuracy [15]. Imaging now plays a pivotal role not only for evaluation of MR etiology and grading, but also in procedural planning, intra-procedural guidance, and post-interventional follow-up, including the detection of repair failure and device dysfunction [16]. At the same time, optimal management increasingly depends on a multidisciplinary Heart Team, integrating cardiologists, imagers, interventionalists, surgeons, anesthesiologists, and intensivists. This collaborative model enhances patient selection, optimizes perioperative safety, and ensures structured long-term follow-up, which has cardiovascular imaging at its center [17]. MVD exemplifies a rapidly evolving field in which improved understanding of disease mechanisms—including the distinction between v-FMR and a-FMR—together with advances in imaging and the rise in surgical, minimally invasive, and transcatheter therapies, has profoundly changed clinical practice.

This review focuses specifically on the role of echocardiography in the pre-procedural assessment of MR, with the aim of guiding the selection of the most appropriate therapeutic strategy for the right patient at the right time.

## 2. Therapeutic Approaches to Mitral Regurgitation: What the Imager Needs to Know

Over the past decades, the therapeutic management of MR has undergone a substantial transformation, evolving from an exclusively surgical domain to a comprehensive framework in which surgical and transcatheter interventions coexist within a patient-tailored continuum of care [18]. For the cardiovascular imager, this shift entails not only an in-depth understanding of valvular anatomy and function, but also the capacity to anticipate and delineate how imaging informs therapeutic selection and guides intra-procedural decision-making [19].

In the surgical arena, the traditional Carpentier philosophy of DMR repair relied on leaflet resection, particularly quadrangular or triangular resections of the posterior leaflet, as the cornerstone of treatment [20]. Although surgical techniques have evolved, the Carpentier functional classification remains crucial because its practical, mechanism-based approach links leaflet motion to underlying etiology and thus guides therapy. More recently, however, a “respect rather than resect” principle has emerged, favoring chordal replacement and leaflet preservation to maintain mobility, minimize annular distortion, and preserve LV geometry whenever befitting [21]. Comparative data suggest that both strategies can achieve durable results with similar impact on the LV [22]. The current emphasis is therefore less on demonstrating the superiority of one approach over another, and more on tailoring the repair to the specific leaflet pathology and the surgeon’s expertise. Within this context, imaging assumes a crucial role by elucidating the underlying mechanisms of MR and furnishing the anatomical details necessary to tailor the reparative approach.

While in DMR, surgical repair offers clear advantages in terms of survival, freedom from reoperation, and valve-related complications, the debate between repair and replacement remains lively in FMR, where a more complex picture has risen: v-FMR has shown higher recurrence rates after repair compared with replacement, even though survival outcomes are similar, whereas a-FMR often benefits from a surgical strategy that addresses atrial pathology (such as with surgical atrial fibrillation ablation) alongside annuloplasty [8,23]. Failures after surgical repair—whether early, due to technical complications such as dehiscence or systolic anterior motion (SAM), or late, related to progressive ventricular or atrial remodeling—underscore the critical role of imaging in anticipating recurrence and guiding timely management [24].

Parallel to surgical evolution, transcatheter therapies have gained increasing relevance. TEER, pioneered with MitraClip, has become the most widely adopted transcatheter approach and has proven particularly effective in v-FMR, especially in patients with disproportionate MR where outcomes are significantly improved [12]. Recent findings from the RESHAPE-HF2 trial indicated that patients with less pronounced MR may also experience favorable outcomes with TEER, in the absence of excessive LV dilatation [25]. Furthermore, current evidence demonstrates that, in this setting, TEER achieves results equivalent to surgical intervention, extending this finding also to patients with merely mild-to-moderate LV dysfunction [26]. The imager plays a central role here, as 3D TEE is fundamental in guiding the interatrial septal puncture, orienting the catheter toward the origin of the regurgitant jet, and providing “en-face” visualization of the valve to optimize clip placement [27]. Advanced techniques such as speckle-tracking echocardiography further enrich this evaluation by quantifying remodeling and refining risk stratification, thereby helping to define the optimal timing for intervention [28,29]. For patients with anatomies unsuitable for TEER and at high surgical risk, TMVR has emerged as a promising option, offering predictable elimination of MR, though still limited by device design, procedural complexity, and questions about long-term durability [30].

The choice between surgical repair or replacement, TEER or TMVR depends on a precise assessment of valve anatomy, ventricular and atrial remodeling that puts the cardiovascular imager at the center, and patient-specific risk. Imaging is pivotal not only in diagnosis but in shaping the therapeutic plan, guiding interventions in real time, and predicting long-term outcomes. For this reason, the modern imager is not just a diagnostician but an active partner in the therapeutic journey of patients with MVD [19].

## 3. Imaging Mitral Regurgitation: From Basics to Frontiers

Imaging plays a central role in the diagnosis, quantification, and management of MR. Echocardiography remains the cornerstone, providing essential information on anatomy, mechanism, and severity. However, the limitations of conventional 2D assessment have progressively driven the integration of advanced modalities, including 3D echocardiography, speckle-tracking echocardiography, CMR, and CT. The multimodality imaging approach has not only improved diagnostic accuracy but also expanded the role of imaging into procedural planning, intra-procedural guidance, and structured follow-up, ultimately shaping therapeutic decision-making [16].

### 3.1. Conventional 2D Echocardiography

**Figure 2:**
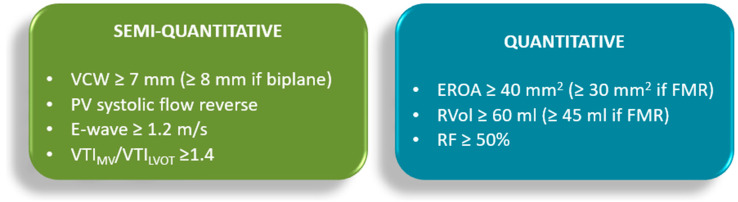
Semi-quantitative (green box) and quantitative (blue box) threshold for severe MR. For further detail, please see Table 1. EROA: effective regurgitant orifice area; E-wave: early diastolic transmitral flow velocity; FMR: functional mitral regurgitation; LVOT: left ventricular outflow tract; MV: mitral valve; PV: pulmonary vein; RF: regurgitant fraction; RVol: regurgitant volume; VCW: vena contracta width; VTI_LVOT: velocity–time integral of the LV outflow tract; VTI_MV: velocity–time integral of the mitral valve.

**Figure 3:**
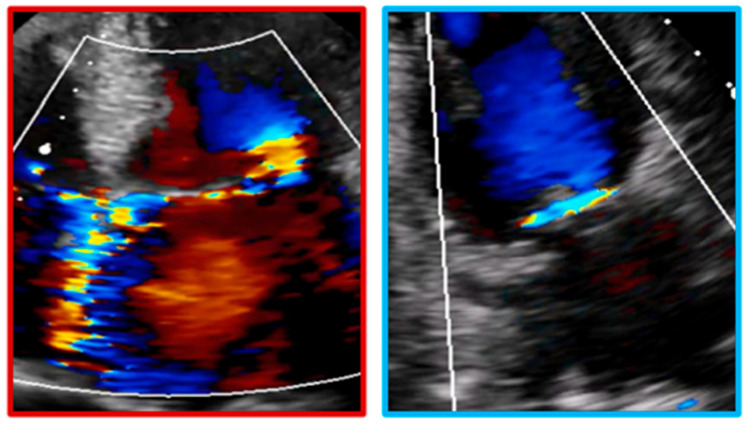
Representation of the “Coanda Effect” and “splay sign”. The first (red panel—Coanda Effect) is displayed in an A4Ch view with an eccentric jet directed towards the interatrial septum. The second (blue panel—splay sign) is shown in an apical two-chamber view, just above the coaptation zone.

Transthoracic echocardiography (TTE) is the primary modality for MR quantification, providing different parameters, conventionally distinguished as qualitative (flow convergence zone and continuous Doppler signal), semi-quantitative [vena contracta width (VCW), vena contracta area (VCA), pulmonary vein flow and mitral to aortic velocity time integral (VTI) ratio], quantitative [regurgitant volume (RVol), effective regurgitant orifice area (EROA) and regurgitant fraction (RF)] and structural (LA/LV size and pulmonary pressure) parameters [15]. The echocardiographic assessment of MR severity is based on a multiparametric and probabilistic approach, in which qualitative, semi-quantitative, and structural parameters may be sufficient to suggest a diagnosis of either mild or severe MR. However, in cases that do not clearly fall into these categories—namely, moderate MR—the integration of quantitative measures (Table 1, Figure 2) becomes essential, as it may allow reclassification into a different severity grade, with significant implications for clinical management and therapeutic decision-making. Moreover, two qualitative aspects deserve mention, as they are commonly associated with severe MR: the Coanda effect and the splay sign. The “Coanda effect” has been extensively described and represents a physical phenomenon whereby an eccentric fluid jet, such as MR traversing the anatomic regurgitant orifice, instead of flowing centrally, tends to follow the curvature of the left atrial wall. The “splay sign”, a relatively underrecognized color Doppler finding, is defined by horizontal dispersion of the color Doppler signal on the atrial side of the mitral valve and is commonly observed in severe MR (Figure 3) [31].

Transesophageal echocardiography (TEE) is not routinely required for quantification, but it is invaluable in defining the mechanism and the site of regurgitation, in assessing the interatrial septum for pre-procedural planning of transcatheter interventions and is often required for the assessment of parameters such as VCA and pulmonary vein flow, which may be sub-optimally visualized with transthoracic imaging [32,33,34].

**Figure 4:**
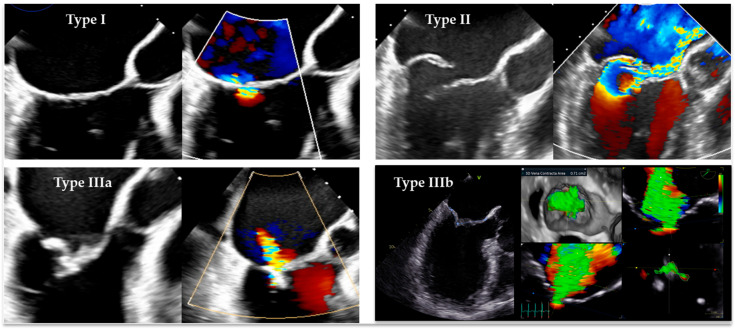
2D-TEE mid-esophageal long-axis views with and without color Doppler, illustrating the mechanisms of MR according to Carpentier’s classification (**Types I**–**III**). (**Type I**): a-FMR due to annular dilatation, characterized by a posterior deviation of the hinge point of the posterior mitral leaflet and mitral annulus toward the outside of the LV crest; the regurgitant jet is usually directed centrally or posteriorly. (**Type II**): flail of the posterior mitral leaflet leading to an eccentric MR jet directed opposite to the affected leaflet. (**Type IIIa**): MR due to rheumatic valve disease with diastolic doming of the anterior leaflet and superimposed calcification of the mid and distal leaflets’ portions. (**Type IIIb**): v-FMR due to apical tethering of mitral leaflets secondary to ischemic LV dilatation and dysfunction.

According to Carpentier’s classification, the mechanisms of MR are traditionally divided into three groups. Type I includes regurgitation caused by annular dilatation/dysfunction (a-FMR) or leaflet alteration (e.g., perforation). Type II is characterized by excessive leaflet motion, such as prolapse/flail or SAM. Type III is subdivided into restricted leaflet motion during diastole (Type IIIa, typically seen in rheumatic mitral valve disease) and restricted motion during systole (Type IIIb, commonly represented by v-FMR) (Figure 4).

Thus, in MR, TTE represents the reference for severity grading, while TEE complements it by providing mechanistic and procedural insights [34].The quantification of MR remains a major challenge. Despite decades of progress, no single echocardiographic parameter has proven sufficiently robust and reproducible to serve as a standalone reference standard. This limitation is reflected in current guideline recommendations, which mandate an integrative multiparametric approach combining the qualitative, semi-quantitative and quantitative measures together with structural parameters (LV and LA dimensions and pulmonary artery pressure) [15].

The need to combine multiple parameters—rather than relying on a single “gold-standard” measure—highlights a fundamental gap in our current diagnostic capabilities. Advanced modalities such as 3D echocardiography and CMR are being increasingly integrated to improve reproducibility and prognostic accuracy, but 2D multiparametric assessment remains the clinical reference endorsed by both European and American guidelines [15,35].

In DMR, 2D echocardiography is highly effective for identifying leaflet prolapse, flail segments, or chordal rupture, which are essential findings for surgical planning and prognostic assessment, as long as the anatomical measurements of the mitral annulus and the risk estimation of SAM are considered (Table 2 and Table 3) [34].

Quantitative indices such as EROA and RVol are widely used, but all have limitations that must be acknowledged. Even if they show a strong correlation with outcomes, substantial inter-observer variability exists [39,40]. RF, particularly when calculated by volumetric methods, has emerged as a more dependable index of MR severity, being relatively independent of LV dimensions compared with RVol [41]. Parameters that depend on the VTI of the regurgitant jet—EROA in the pulsed-wave Doppler or volumetric methods, and all three indices (EROA, RVol and RF) when using the PISA method—may be prone to under-estimation due to difficulty in aligning with eccentric jets [42]. Moreover, the duration of the regurgitant jet (holosystolic vs. non-holosystolic) critically affects the calculated RVol: two patients with the same EROA and flow rate may have different regurgitant burdens de-pending on jet duration, and it is duration—not EROA—that best stratifies prognosis in prolapse-related MR [43]. Another pitfall of the PISA method is that the so-called “prolapse volume”—the regurgitant component due to leaflet billowing—is not accounted for in the formula, further leading to underestimation in degenerative disease [44].

**Table 3:** Complementary echocardiographic measurements for mitral valve repair. TA, tricuspid annulus; AP, antero-posterior * [45].

VIEW	MEASUREMENT	CYCLE	CUT-OFF	AIM
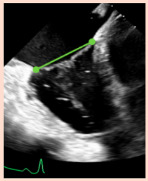	TA diameter	End-diastole	≥40 mm or >21 mm/m^2^	Tricuspid annuloplasty
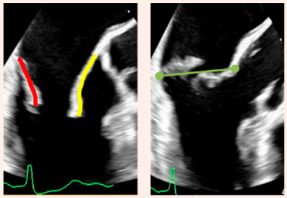	AP/(AL + PL) AP/AL	Diastole Early-mid systole	>0.7 * >1.4 *	Need for annuloplasty

In FMR, the complexity increases further: the regurgitant orifice is dynamic and often non-circular, challenging the geometric assumptions of 2D echocardiography and making the accuracy of those indices debatable [46]. Moreover, MR severity is heavily load-dependent, and hemodynamic changes—such as those induced by anesthesia—may lead to significant underestimation of the true regurgitant burden when assessed under resting conditions, exacerbating intra- and inter-patient variability [47].

In v-FMR, quantification is even more challenging. Beyond the non-circular geometry of the regurgitant orifice, EROA and RVol are strongly influenced by LV end-diastolic volume and LV ejection fraction (LVEF), such that the same RVol may represent different severities depending on LV function or systemic pressures. Similarly, at the same LVEF, an identical EROA may or may not be prognostically significant depending on loading conditions [48]. This may explain why discordance among methods is common in v-FMR [49]. Importantly, in advanced disease stages, typical of the MITRA-FR (Percutaneous Repair or Medical Treatment for Functional Mitral Regurgitation) population, MR often reflects an epiphenomenon of progressive LV dilatation rather than a primary driver of symptoms, in contrast to the COAPT (Cardiovascular Outcomes Assessment of the MitraClip Percutaneous Therapy for Heart Failure Patients with Functional Mitral Regurgitation) population, which shows an earlier stage of disease, where MR is the main determinant of clinical status [48]. Lastly, in the setting of FMR, EROA and RVol calculated by volumetric methods and not by PISA seem to show greater prognostic value [49].

In a-FMR, the accurate assessment of MR severity is hampered by several limitations. First, subtle annular dilatation and malcoaptation may be underestimated with echocardiography. Second, the presence of rapid or extremely irregular R-R intervals may vitiate the accurate measurement of LV and LA volumes, so much so that the beat-to-beat variation in stroke volume (up to 16–28%) makes the volumetric method unreliable. Third, the PISA method is challenged by intra- and inter-beat variability of the regurgitant orifice area and by the frequent elliptical shape of the orifice, which violates the geometric assumption of the technique, as in v-FMR. Fourth, indirect indices such as pulmonary venous flow are confounded by AF itself or by elevated LA pressure due to atrial stiffness, reducing their specificity for MR. Moreover, current thresholds proposed for FMR were derived from populations with v-FMR [50]. This underscores that existing cut-offs may fail to capture the true hemodynamic burden of a-FMR and explains the growing interest in LA strain and 3D imaging to improve risk stratification in this subgroup [51,52].

Finally, limitations common to all MR forms should be acknowledged. LV volumes can be underestimated due to 2D foreshortening, affecting both volumetric calculations and RF estimation [53,54]. The PISA method is inconsistent in the presence of multiple jets and, being a single-frame measurement, fails to account for the dynamic nature of MR throughout systole [41,55]. Most importantly, MR grading still relies on arbitrary historical thresholds, in particular regarding DMR, that do not account for patient-specific variability: the same RVol may have very different clinical significance depending on sex, body size, chamber compliance, ventricular function and volumes, just as the determination of a unique standardized RVol threshold for severe MR may be questionable [41,48,56].

In summary, while 2D echocardiography is indispensable for mechanistic classification and initial quantification, its methodological and physiological limitations—both in DMR and in FMR—have increasingly redirected research efforts toward advanced imaging modalities such as 3D echocardiography, CMR and speckle-tracking analysis, which may offer incremental value for risk stratification and therapeutic guidance [57].

### 3.2. Advanced Echocardiography and Other Imaging Modalities

**Figure 5:**
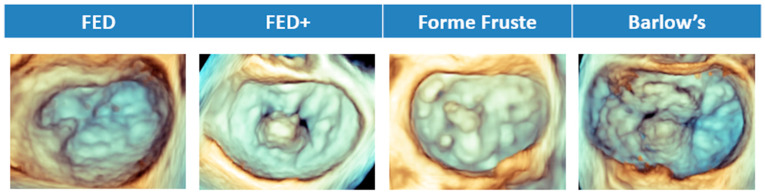
3DTEE Representation of Degenerative Mitral Valve Disease Phenotypes. Fibroelastic deficiency (FED) is defined by thin valve leaflets and often presents with a localized flail resulting from chordal rupture. In contrast, FED-plus (FED+) originates as classical FED but, over time, the chronic prolapse leads to secondary myxomatous alterations with leaflet thickening and enlargement. Forme fruste refers to a degenerative condition characterized by myxomatous changes and excess tissue, typically involving more than one leaflet segment, but without marked valve enlargement—thereby differentiating it from Barlow’s disease. In Barlow’s disease, the defining features are a markedly enlarged valve with diffuse myxomatous remodeling, abundant leaflet tissue, and chordae that are thickened, elongated, and frequently ruptured.

3D echocardiography has transformed the assessment of MV anatomy and MR. Compared with 2D, it provides direct planimetry of the regurgitant orifice, also known as VCA, a more accurate quantification of annular dimensions, and precise localization of leaflet lesions [58]. 3D enables a more accurate distinction of the four phenotypes of MV pathology underlying DMR—fibroelastic deficiency (FED), FED plus, forme fruste, and Barlow’s disease (Figure 5)—compared with conventional 2D and improves surgical planning by defining the extent and location of prolapse or flail [59]. Moreover, 3D-RF demonstrated superior discriminatory power compared with 2D-RF in differentiating clinically significant MR, just as 3D-RVol provides MR grades in closer agreement with CMR, outperforming conventional PISA-derived measurements [60,61].

In FMR, 3D echocardiography enhances the characterization of underlying mechanisms of functional and ischemic regurgitation, while providing more accurate quantification, with excellent agreement with CMR compared to conventional 2D [58,62].

In the setting of TMVR, comprehensive 3D-TEE has proven feasible and reliable as an alternative to CT for pre-procedural planning, providing accurate assessment of annular geometry and enabling prediction of LVOT obstruction risk [63].

Advanced echocardiographic techniques, including 3D imaging and speckle-tracking echocardiography, can enhance the evaluation of cardiac remodeling in MR, thereby refining risk stratification and helping to optimize the timing of interventions. These advanced techniques represent a valuable tool for refining risk stratification in patients with MR. They are essential not only for evaluating the valve itself but also for detecting extra-mitral valve cardiac damage, including left atrial dilation, left ventricular dilation and dysfunction, pulmonary hypertension, right ventricular dysfunction, concomitant tricuspid regurgitation, and systemic congestion. These conditions are associated with worse prognosis and should be carefully considered when planning intervention [64]. For example, significant tricuspid annular dilation or concomitant tricuspid regurgitation may indicate the need to plan a concomitant tricuspid annuloplasty alongside the mitral intervention [13]. Speckle-tracking echocardiography enables sensitive detection of subclinical LV dysfunction. LV global longitudinal strain (GLS) predicts adverse outcomes in DMR even with preserved LV ejection fraction [65]. In a COAPT-like population, LV-GLS served as powerful predictors of outcomes [28,66]. LA strain reflects atrial compliance and remodeling and has proven to be a robust marker for predicting clinical outcomes and functional capacity in patients with severe DMR [67]. Right ventricular free-wall and GLS have emerged as independent predictors of adverse outcomes in patients with FMR undergoing TEER, and in patients with DMR undergoing surgical MV repair [29,68].

In asymptomatic patients with moderate-to-severe DMR and preserved LVEF, exercise stress echocardiography can unmask latent LV dysfunction, reveal the dynamic worsening of regurgitation, resolve discrepancies between symptoms and resting findings, and provide incremental value for immediate risk stratification and optimal timing of surgery [69]. In patients with FMR, exercise echocardiography is particularly valuable for unmasking disproportionate exertional dyspnoea, clarifying unexplained episodes of acute pulmonary edema, assessing moderate MR before surgical revascularization and refining individual risk stratification [70,71].

CMR ensures highly reproducible quantification of MR and delivers powerful prognostic insights [72]. Beyond quantification, the detection of myocardial fibrosis has been linked to adverse outcomes and may refine patient selection [73].

CT is pivotal for pre-procedural assessment in TMVR, allowing accurate measurement of annular size, extension of annular calcification, and LVOT geometry to predict the risk of obstruction [74]. CT is also used in surgical planning, particularly in reoperation or when annular calcification is present, and increasingly supports simulation and 3D printing for device sizing.

### 3.3. Imaging for Procedural Guidance and Follow-Up

**Figure 6:**
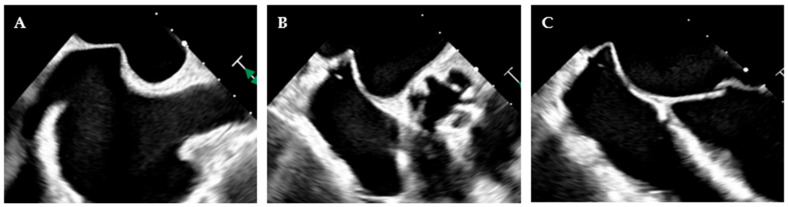
TEE guidance of transseptal puncture in multiple projections. (**A**) Mid-esophageal bicaval-view showing the supero-inferior axis of the interatrial septum and fossa ovalis. (**B**) Mid-esophageal aortic valve short axis view, showing the antero-posterior axis of the interatrial septum and fossa ovalis. (**C**) Mid-esophageal four-chamber view, allowing accurate quantification of height from annulus plane. Note that a good transeptal puncture must be in a central-superior and posterior area of the fossa ovalis, ensuring adequate height from the annulus plane.

The role of imaging in MVD extends far beyond diagnosis and quantification. As surgical, minimally invasive, and transcatheter therapies have evolved, imaging has become a central component of procedural planning, intra-procedural navigation, and long-term follow-up [75]. Real-time 3D-TEE has become indispensable for guiding mitral interventions and choosing the best strategy for the patient [27]. For surgical MV repair and specifically for minimally invasive MV repair, TEE and 3D-TEE are fundamental, as they guide the surgeon in targeting the pathological area, selecting the appropriate annular size, and identifying intraoperative complications such as systolic anterior mitral leaflet motion causing LVOT obstruction. When such issues arise, they should be promptly addressed by choosing the most appropriate surgical solution. In TEER, TEE is fundamental to guide the interatrial septal puncture and to orient the catheter toward the origin of the regurgitant jet, ensuring precise targeting of the valve pathology (Figure 6). Moreover, it provides an “en-face” visualization of the MV, which enables optimal clip orientation and improves the effectiveness of the repair. In TMVR, it supports accurate valve positioning, detection of paravalvular leaks, and assessment of LVOT obstruction risk and occurrence.

Compared with 2D, 3D TEE reduces operator variability and enhances procedural success, explaining why it is now considered the standard of care in high-volume centers [76].

Post-procedural imaging is essential for early detection of device and prosthesis dysfunction or MR recurrence. After surgical MV repair and TEER, the immediate intra-operative result is verified by TEE evaluation, whereas TTE remains the reference for assessing residual regurgitation, transmitral gradient, and LV remodeling during follow-up, where TEE is reserved for cases in which additional information is required [13,77]. Similar considerations apply to TMVR, where TEE—or alternatively CT—is indicated to address uncertainties left unresolved by TTE during follow-up [16]. Importantly, both v-FMR and a-FMR may recur over time due to ongoing LA or LV remodeling, underscoring the importance of structured imaging follow-up.

### 3.4. Emerging Frontiers in Imaging

**Figure 7:**
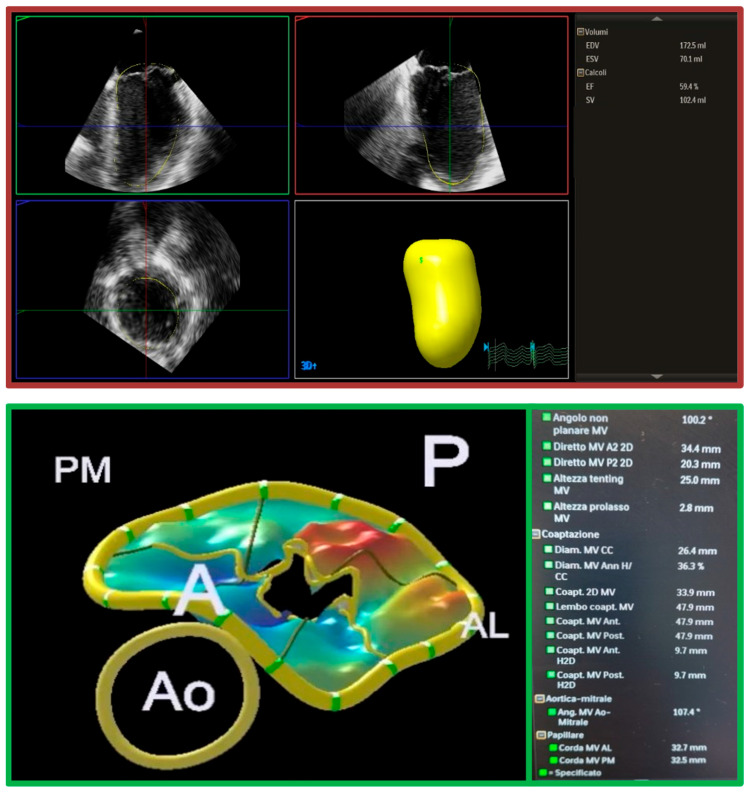
The 3DQA and MVN tools. The 3DQA tool (red panel) is a semi-automated and guided tool that provides the 3D quantification of LV volumes, stroke volume and ejection fraction from a 3D dataset. The MVN tool (green panel) is a guided tool that facilitates the acquisition of comprehensive anatomical measurements and calculations related to the mitral valve apparatus. 3D: three-dimensional; A: anterior; AL: anterolateral; Ao: aorta; EDV: end-diastolic volume; EF: ejection fraction; ESV: end-systolic volume; MV: mitral valve; P: posterior; PM: posteromedial; SV: stroke volume.

Artificial intelligence (AI) and machine learning are increasingly applied to automate MR quantification, mechanism definition, automatic MV measurements and LV/LA volume assessment, and risk prediction (Figure 7) [78]. Automated algorithms have shown promise in reducing inter-observer variability, improving reproducibility, and shortening analysis time [79]. Early applications also suggest a role for AI in predicting outcomes after transcatheter or surgical MV repair [80,81,82]. The potential for AI-driven real-time decision support during interventions represents an exciting step toward personalized imaging-guided therapy.

The application of fusion imaging—such as echocardiography/fluoroscopy, CT/fluoroscopy, and echocardiography/CT—has transformed how operators visualize the cardiac structures, improving precision, safety, and procedural success [16,83,84].

The integration of fluoroscopy with echocardiography may provide continuous spatial orientation facilitating device positioning, in particular in the presence of challenging MV anatomy for percutaneous repair [85]. The combination of CT-derived reconstructions with live fluoroscopy or echocardiography offers further benefits for TMVR planning and implantation, particularly in anatomically complex patients [83].

Advances in virtual and augmented reality have enabled immersive visualization of valve anatomy, offering new opportunities in operator training and procedural rehearsal [86]. Patient-specific 3D printing of MV and surrounding anatomy has gained traction as a tool for pre-procedural planning, particularly for TMVR in complex anatomies or heavily calcified annuli [87]. These technologies allow simulation of device deployment and prediction of complications such as LVOT obstruction, enhancing pre-interventional understanding, especially in anatomically challenging scenarios.

Taken together, these emerging technologies illustrate how imaging is evolving into a dynamic, interactive partner in therapy. AI promises automated, patient-specific quantification; 3D printing and virtual reality provide unprecedented anatomical insight; and fusion platforms enable real-time multimodal guidance [88]. Their integration into clinical practice will likely redefine procedural planning and execution, potentially bridging the gap toward precision medicine in MVD.

## 4. Conclusions

Echocardiography is the cornerstone of MR management, providing not only diagnosis but also a detailed understanding of the mechanism underlying valve dysfunction. Beyond the classification in DMR and FMR and accurate quantification, advanced imaging modalities such as 3D and strain imaging may refine risk stratification and offer reliable prognostic information. Careful assessment of ventricular and atrial remodeling, pulmonary pressures, and right heart function allows treatment to be planned before irreversible damage occurs. Moreover, echocardiography has become a decisive tool in therapy selection: in surgery, it guides repair strategies, while in transcatheter interventions, it is essential for trans-septal puncture, catheter orientation, and real-time confirmation of procedural success. In essence, echocardiography has evolved from a diagnostic technique into the central enabler of precision treatment in MR, ensuring that the most appropriate therapy is delivered to the right patient at the right time.

## Figures and Tables

**Table 1:** Summary of semi-quantitative (green box) and quantitative criteria (blue box) for MR severity assessment and corresponding measurement methods. VCW, vena contracta width; VTI, velocity time integral; PWD, pulsed-wave Doppler; A4Ch, apical four chamber; LVOT, left ventricular outflow tract; EROA, effective regurgitant orifice area; PISA, proximal isovelocity surface area; PISAr, PISA radium; PV, pulmonary vein; Va, velocity of aliasing; Reg Flow, regurgitant flow; CWD, continuous wave Doppler; Vmax, maximum velocity; RVol, regurgitant volume; RF, regurgitant fraction; SV_MV_, stroke volume across mitral valve; SV_LV_, left ventricular stroke volume; MA, mitral annulus; EDV, end-diastolic volume; ESV, end-systolic volume; PLAX, parasternal long-axis. ^†^ Usually, the mean value between A4Ch and two-chamber view. * Systolic reverse may also arise from other causes (e.g., AF or elevated LA pressure). ^‡^ Pay attention to alternative conditions of high E-wave peak (e.g., diastolic disfunction or mitral stenosis).

**SEMIQUANTITATIVE**	
**VCW (mm)** ^†^	Visualize the three components—flow convergence, vena contracta and distal jet expansion—of the MR preferably in a view where the ultrasound beam is parallel to the vena contracta diameter; the vena contracta width represents the narrowest diameter of the regurgitant jet, located between the flow convergence zone and the region of distal jet expansion and lies downstream of the anatomic regurgitant orifice.
**PV flow** *	Acquire the PWD signal of pulmonary vein flow by placing a small sample volume (3–5 mm) just 1 cm inside the pulmonary vein; TEE is generally required to adequately sample all pulmonary veins, as systolic reverse may be confined to a single vein only.
**Mitral inflow** ^‡^	Acquire the PWD signal of transmitral flow by placing the sample volume at the level of MV leaflets’ tips using A4Ch view.
**VTI_MV_/VTI_LVOT_**	It is the ratio between the VTI of mitral inflow (VTI_MV_) and the VTI of LVOT (VTI_LVOT_). The former can be obtained as described above, the latter by placing the PWD sample volume at the level of LVOT.
**QUANTITATIVE**	
**EROA (mm^2^)**	*PISA method:* - *Step 1*: measure the PISAr from the A4Ch view using a mid-systolic frame, after reducing the Nyquist limit (Va) to 20–40 cm/s to clearly delineate the hemispheric contour. The RegFlow is then calculated as: RegFlow = 2π × (PISAr)^2^ × Va. - *Step 2*: acquire the CWD signal of the regurgitant jet to measure both the VTI_MR_ and the Vmax of the MR to obtain the EROA (=Reg Flow/Vmax) and the RVol (=EROA × VTI_MR_), respectively. - *Step 3*: calculate the RF using the SV_MV_ or, alternatively, the SV_LV_. The first is obtained as SV_MV_ = VTI_MA_ × MAd, where VTI_MA_ is measured by PWD at the MA level (not at the leaflet tips), and MAd is the annular diameter (preferably in a biplane view or in A4Ch if biplane is not available). The second can be calculated as: SV_LV_ = EDV_LV_ − ESV_LV_. RF can be expressed as: RF = RVol/ × 100 or RF = RVol/SV_LV_ × 100. *PWD method:* - *Step 1*: calculate the SV_MV_, as previously described, and the SV_LVOT_ as follows: SV_LVOT_ = VTI_LVOT_ × LVOTd, where LVOTd is the diameter of LVOT, measured in PLAX view. - *Step 2*: calculate the RVol as: RVol = SV_MV_ − SV_LVOT_. Calculate the EROA as: EROA = RVol/VTI_MR_. Calculate the RF as: RVol/SV_MV_ × 100. *Volumetric method* - *Step 1*: calculate SV_LV_ and SV_LVOT_ as described above. - *Step 2*: calculate RVol as follows: RVol = SV_LV_ − SV_LVOT_. - *Step 3*: calculate EROA and RF% as described in PWD method.
**RVol (mL)**	
**RF (%)**	

**Table 2:** Echocardiographic measurements guiding mitral valve repair: cut-offs and clinical implications. * Choose the frame that provides the best view of the leaflets in their entirety, from the hinge point at the annulus level to the leaflet tip. AP, antero-posterior; MVA, mitral valve area; AL, anterior leaflet; PL, posterior leaflet; EDD, end-diastolic diameter; IVS, interventricular septum; C-sept, coaptation-septum. ^†^ [36]. ^‡^ [37]. ^#^ [38].

VIEW	MEASUREMENT	CYCLE	CUT-OFF	AIM
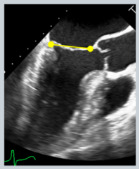	AP diameter	End-systolic	>35 mm	Annulus dilatation (Ring size)
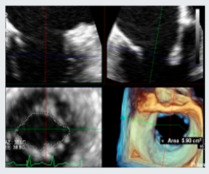	MVA	Diastole	nd	Risk of MS
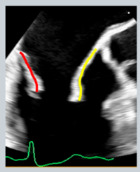	Al length PL length	*	>20 mm >15 mm	Ring size Risk of SAM
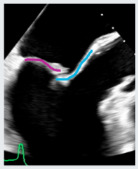	AL/PL ratio	Mid-systole	≤1.3 ^†^	Risk of SAM
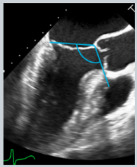	Ao-MV angle	Mid-systole	<120° ^‡#^	Risk of SAM
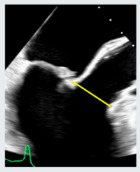	C-sept distance	Mid-systole	<25 mm ^‡^	Risk of SAM
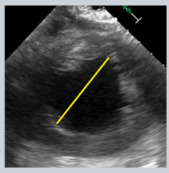	EDD	End-diastole	<45 mm ^‡^	Risk of SAM
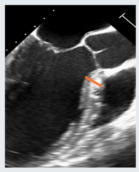	Basal IVS	End-diastole	≥15 mm ^‡^	Risk of SAM

## Data Availability

No new data were created or analyzed in this study. Data sharing is not applicable to this article.

## Abbreviations

The following abbreviations are used in this manuscript:

2D	Bidimensional
3D	Three-dimensional
a-FMR	Atrial Functional Mitral Regurgitation
AF	Atrial Fibrillation
AI	Artificial Intelligence
AR	Augmented Reality
CMR	Cardiac Magnetic Resonance
CT	Computed Tomography
DMR	Degenerative Mitral Regurgitation
EROA	Effective Regurgitant Orifice Area
FED	Fibroelastic Deficiency
FMR	Functional Mitral Regurgitation
v-FMR	Ventricular Functional Mitral Regurgitation
GLS	Global Longitudinal Strain
LA	Left Atrial/Left Atrium
LV	Left Ventricular/Left Ventricle
LVEF	Left Ventricular Ejection Fraction
LVOT	Left Ventricular Outflow Tract
MVD	Mitral Valve Disease
MR	Mitral Regurgitation
MV	Mitral Valve
PISA	Proximal Isovelocity Surface Area
RF	Regurgitant Fraction
RVol	Regurgitant Volume
SAM	Systolic Anterior Motion
TEE	Transesophageal Echocardiography
TEER	Transcatheter Edge-To-Edge Repair
TMVR	Transcatheter Mitral Valve Replacement
TTE	Transthoracic Echocardiography
v-FMR	Ventricular Functional Mitral Regurgitation
VCA	Vena Contracta Area
VCW	Vena Contracta Width
VR	Virtual Reality
VTI	Velocity Time Integral

## References

[1] Silbiger J.J. (2011). Mechanistic Insights into Ischemic Mitral Regurgitation: Echocardiographic and Surgical Implications. J. Am. Soc. Echocardiogr..

[2] Zoghbi W.A., Levine R.A., Flachskampf F., Grayburn P., Gillam L., Leipsic J., Thomas J.D., Kwong R.Y., Vandervoort P., Chandrashekhar Y. (2022). Atrial Functional Mitral Regurgitation. JACC Cardiovasc. Imaging.

[3] Farhan S., Silbiger J.J., Halperin J.L., Zhang L., Dukkipati S.R., Vogel B., Kini A., Sharma S., Lerakis S. (2022). Pathophysiology, Echocardiographic Diagnosis, and Treatment of Atrial Functional Mitral Regurgitation. J. Am. Coll. Cardiol..

[4] Okamoto C., Okada A., Nishimura K., Moriuchi K., Amano M., Takahama H., Amaki M., Hasegawa T., Kanzaki H., Fujita T. (2021). Prognostic comparison of atrial and ventricular functional mitral regurgitation. Open Heart.

[5] Coffey S., Roberts-Thomson R., Brown A., Carapetis J., Chen M., Enriquez-Sarano M., Zühlke L., Prendergast B.D. (2021). Global epidemiology of valvular heart disease. Nat. Rev. Cardiol..

[6] So K.C.-Y., Yap J., Song G.-Y., Poon K., Sung S.H., Chandavimol M., Hayashida K., Park D.-W., Ewe S.-H., Chen M. (2025). Epidemiology of Valvular Heart Disease in Asia Pacific Region. JACC Asia.

[7] Lazam S., Vanoverschelde J.L., Tribouilloy C., Grigioni F., Suri R.M., Avierinos J.F., de Meester C., Barbieri A., Rusinaru D., Russo A. (2017). Twenty-Year Outcome After Mitral Repair Versus Replacement for Severe Degenerative Mitral Regurgitation. Circulation.

[8] Acker M.A., Parides M.K., Perrault L.P., Moskowitz A.J., Gelijns A.C., Voisine P., Smith P.K., Hung J.W., Blackstone E.H., Puskas J.D. (2014). Mitral-Valve Repair versus Replacement for Severe Ischemic Mitral Regurgitation. N. Engl. J. Med..

[9] Davierwala P.M., Seeburger J., Pfannmueller B., Garbade J., Misfeld M., Borger M.A., Mohr F.W. (2013). Minimally invasive mitral valve surgery: “The Leipzig experience”. Ann. Cardiothorac. Surg..

[10] Feldman T., Wasserman H.S., Herrmann H.C., Gray W., Block P.C., Whitlow P., St Goar F., Rodriguez L., Silvestry F., Schwartz A. (2005). Percutaneous Mitral Valve Repair Using the Edge-to-Edge Technique. J. Am. Coll. Cardiol..

[11] Feldman T., Foster E., Glower D.D., Kar S., Rinaldi M.J., Fail P.S., Smalling R.W., Siegel R., Rose G.A., Engeron E. (2011). Percutaneous Repair or Surgery for Mitral Regurgitation. N. Engl. J. Med..

[12] Stone G.W., Abraham W.T., Lindenfeld J., Kar S., Grayburn P.A., Lim D.S., Mishell J.M., Whisenant B., Rinaldi M., Kapadia S.R. (2023). Five-Year Follow-up after Transcatheter Repair of Secondary Mitral Regurgitation. N. Engl. J. Med..

[13] Praz F., Borger M.A., Lanz J., Marin-Cuartas M., Abreu A., Adamo M., Ajmone Marsan N., Barili F., Bonaros N., Cosyns B. (2025). 2025 ESC/EACTS Guidelines for the management of valvular heart disease. Eur. J. Cardio-Thorac. Surg..

[14] Yousef S., Arnaoutakis G.J., Gada H., Smith A.J.C., Sanon S., Sultan I. (2022). Transcatheter mitral valve therapies: State of the art. J. Card. Surg..

[15] Lancellotti P., Pibarot P., Chambers J., La Canna G., Pepi M., Dulgheru R., Dweck M., Delgado V., Garbi M., Vannan M.A. (2022). Multi-modality imaging assessment of native valvular regurgitation: An EACVI and ESC council of valvular heart disease position paper. Eur. Heart J. Cardiovasc. Imaging.

[16] Agricola E., Ancona F., Bartel T., Brochet E., Dweck M., Faletra F., Lancellotti P., Mahmoud-Elsayed H., Marsan N.A., Maurovich-Hovart P. (2023). Multimodality imaging for patient selection, procedural guidance, and follow-up of transcatheter interventions for structural heart disease: A consensus document of the EACVI Task Force on Interventional Cardiovascular Imaging: Part 1: Access routes, transcatheter aortic valve implantation, and transcatheter mitral valve interventions. Eur. Heart J. Cardiovasc. Imaging.

[17] Antonides C.F.J., Mack M.J., Kappetein A.P. (2017). Approaches to the Role of The Heart Team in Therapeutic Decision Making for Heart Valve Disease. Struct. Heart.

[18] Lio A., Loreni F., Miceli A., Wiedemann D. (2024). Editorial: Evolution of mitral valve disease treatment: From surgery to transcatheter therapy. Front. Cardiovasc. Med..

[19] Little S.H. (2023). Interventional Echocardiography: The Emergence of a New Imaging Specialty. J. Am. Soc. Echocardiogr..

[20] Carpentier A. (1983). Cardiac valve surgery-the “French correction”. J. Thorac. Cardiovasc. Surg..

[21] Dreyfus G.D., Dulguerov F., Marcacci C., Haley S.R., Gkouma A., Dommerc C., Albert A. (2018). “Respect when you can, resect when you should”: A realistic approach to posterior leaflet mitral valve repair. J. Thorac. Cardiovasc. Surg..

[22] van Wijngaarden A.L., Tomšič A., Mertens B.J.A., Fortuni F., Delgado V., Bax J.J., Klautz R.J.M., Marsan N.A., Palmen M. (2022). Mitral valve repair for isolated posterior mitral valve leaflet prolapse: The effect of respect and resect techniques on left ventricular function. J. Thorac. Cardiovasc. Surg..

[23] Wagner C.M., Brescia A.A., Watt T.M.F., Bergquist C., Rosenbloom L.M., Ceniza N.N., Markey G.E., Ailawadi G., Romano M.A., Bolling S.F. (2024). Surgical strategy and outcomes for atrial functional mitral regurgitation: All functional mitral regurgitation is not the same!. J. Thorac. Cardiovasc. Surg..

[24] Moore R.A., Wierup P., Tappuni S., Houghtaling P.L., Burns D.J.P., Chemtob R., Blackstone E.H., Svensson L.G., Gillinov A.M. (2024). Reoperation after early and late failure of mitral valve repair for degenerative disease. J. Thorac. Cardiovasc. Surg..

[25] Anker S.D., Friede T., von Bardeleben R.S., Butler J., Khan M.S., Diek M., Heinrich J., Geyer M., Placzek M., Ferrari R. (2024). Transcatheter Valve Repair in Heart Failure with Moderate to Severe Mitral Regurgitation. N. Engl. J. Med..

[26] Baldus S., Doenst T., Pfister R., Gummert J., Kessler M., Boekstegers P., Lubos E., Schröder J., Thiele H., Walther T. (2024). Transcatheter Repair versus Mitral-Valve Surgery for Secondary Mitral Regurgitation. N. Engl. J. Med..

[27] Brugiatelli L., Rolando M., Lofiego C., Fogante M., Capodaglio I., Patani F., Tofoni P., Maurizi K., Nazziconi M., Massari A. (2024). Transcatheter Mitral Valve Intervention: Current and Future Role of Multimodality Imaging for Device Selection and Periprocedural Guidance. Medicina.

[28] Medvedofsky D., Milhorini Pio S., Weissman N.J., Namazi F., Delgado V., Grayburn P.A., Kar S., Lim D.S., Lerakis S., Zhou Z. (2021). Left Ventricular Global Longitudinal Strain as a Predictor of Outcomes in Patients with Heart Failure with Secondary Mitral Regurgitation: The COAPT Trial. J. Am. Soc. Echocardiogr..

[29] Lupi L., Italia L., Pagnesi M., Pancaldi E., Ancona F., Stella S., Pezzola E., Cimino G., Saccani N., Ingallina G. (2023). Prognostic value of right ventricular longitudinal strain in patients with secondary mitral regurgitation undergoing transcatheter edge-to-edge mitral valve repair. Eur. Heart J. Cardiovasc. Imaging.

[30] Hensey M., Brown R.A., Lal S., Sathananthan J., Ye J., Cheung A., Blanke P., Leipsic J., Moss R., Boone R. (2021). Transcatheter Mitral Valve Replacement. JACC Cardiovasc. Interv..

[31] Wiener P.C., Friend E.J., Bhargav R., Radhakrishnan K., Kadem L., Pressman G.S. (2020). Color Doppler Splay: A Clue to the Presence of Significant Mitral Regurgitation. J. Am. Soc. Echocardiogr..

[32] Shanks M., Siebelink H.M.J., Delgado V., van de Veire N.R.L., Ng A.C.T., Sieders A., Schuijf J.D., Lamb H.J., Ajmone Marsan N., Westenberg J.J.M. (2010). Quantitative Assessment of Mitral Regurgitation. Circ. Cardiovasc. Imaging.

[33] Itakura K., Utsunomiya H., Takemoto H., Takahari K., Ueda Y., Izumi K., Ikenaga H., Hidaka T., Fukuda Y., Nakano Y. (2021). Prevalence, distribution, and determinants of pulmonary venous systolic flow reversal in severe mitral regurgitation. Eur. Heart J. Cardiovasc. Imaging.

[34] Gheorghe L.L., Mobasseri S., Agricola E., Wang D.D., Milla F., Swaans M., Pandis D., Adams D.H., Yadav P., Sievert H. (2021). Imaging for Native Mitral Valve Surgical and Transcatheter Interventions. JACC Cardiovasc. Imaging.

[35] Zoghbi W.A., Adams D., Bonow R.O., Enriquez-Sarano M., Foster E., Grayburn P.A., Hahn R.T., Han Y., Hung J., Lang R.M. (2017). Recommendations for Noninvasive Evaluation of Native Valvular Regurgitation: A Report from the American Society of Echocardiography Developed in Collaboration with the Society for Cardiovascular Magnetic Resonance. J. Am. Soc. Echocardiogr..

[36] Maslow A.D., Regan M.M., Haering J.M., Johnson R.G., Levine R.A. (1999). Echocardiographic predictors of left ventricular outflow tract obstruction and systolic anterior motion of the mitral valve after mitral valve reconstruction for myxomatous valve disease. J. Am. Coll. Cardiol..

[37] Varghese R., Itagaki S., Anyanwu A.C., Trigo P., Fischer G., Adams D.H. (2014). Predicting systolic anterior motion after mitral valve reconstruction: Using intraoperative transoesophageal echocardiography to identify those at greatest risk. Eur. J. Cardio-Thorac. Surg..

[38] Mihaileanu S., Marino J.P., Chauvaud S., Perier P., Forman J., Vissoat J., Julien J., Dreyfus G., Abastado P., Carpentier A. (1988). Left ventricular outflow obstruction after mitral valve repair (Carpentier’s technique). Proposed mechanisms of disease. Circulation.

[39] Enriquez-Sarano M., Avierinos J.F., Messika-Zeitoun D., Detaint D., Capps M., Nkomo V., Scott C., Schaff H.V., Tajik A.J. (2005). Quantitative Determinants of the Outcome of Asymptomatic Mitral Regurgitation. N. Engl. J. Med..

[40] Coisne A., Aghezzaf S., Edmé J.L., Bernard A., Ma I., Bohbot Y., Di Lena C., Nicol M., Lavie Badie Y., Eyharts D. (2020). Reproducibility of reading echocardiographic parameters to assess severity of mitral regurgitation. Insights from a French multicentre study. Arch. Cardiovasc. Dis..

[41] Altes A., Levy F., Iacuzio L., Dumortier H., Toledano M., Tartar J., Tribouilloy C., Maréchaux S. (2022). Comparison of Mitral Regurgitant Volume Assessment between Proximal Flow Convergence and Volumetric Methods in Patients with Significant Primary Mitral Regurgitation: An Echocardiographic and Cardiac Magnetic Resonance Imaging Study. J. Am. Soc. Echocardiogr..

[42] Grayburn P.A., Weissman N.J., Zamorano J.L. (2012). Quantitation of Mitral Regurgitation. Circulation.

[43] Topilsky Y., Michelena H., Bichara V., Maalouf J., Mahoney D.W., Enriquez-Sarano M. (2012). Mitral Valve Prolapse With Mid-Late Systolic Mitral Regurgitation. Circulation.

[44] Levy F., Iacuzio L., Marechaux S., Civaia F., Dommerc C., Wautot F., Tribouilloy C., Eker A. (2021). Influence of Prolapse Volume in Mitral Valve Prolapse. Am. J. Cardiol..

[45] Maisano F., La Canna G., Grimaldi A., Viganò G., Blasio A., Mignatti A., Colombo A., Maseri A., Alfieri O. (2007). Annular-to-Leaflet Mismatch and the Need for Reductive Annuloplasty in Patients Undergoing Mitral Repair for Chronic Mitral Regurgitation Due to Mitral Valve Prolapse. Am. J. Cardiol..

[46] Grayburn P.A., Carabello B., Hung J., Gillam L.D., Liang D., Mack M.J., McCarthy P.M., Miller D.C., Trento A., Siegel R.J. (2014). Defining “Severe” Secondary Mitral Regurgitation. J. Am. Coll. Cardiol..

[47] Alachkar M.N., Kirschfink A., Grebe J., Schälte G., Almalla M., Frick M., Schröder J.W., Vogt F., Marx N., Altiok E. (2022). General Anesthesia Leads to Underestimation of Regurgitation Severity in Patients with Secondary Mitral Regurgitation Undergoing Transcatheter Mitral Valve Repair. J. Cardiothorac. Vasc. Anesth..

[48] Grayburn P.A., Sannino A., Packer M. (2019). Proportionate and Disproportionate Functional Mitral Regurgitation: A New Conceptual Framework That Reconciles the Results of the MITRA-FR and COAPT Trials.

[49] Igata S., Cotter B.R., Hang C.T., Morikawa N., Strachan M., Raisinghani A., Blanchard D.G., DeMaria A.N. (2021). Optimal Quantification of Functional Mitral Regurgitation: Comparison of Volumetric and Proximal Isovelocity Surface Area Methods to Predict Outcome. J. Am. Heart Assoc..

[50] Nappi F. (2024). Atrial functional mitral regurgitation in cardiology and cardiac surgery. J. Thorac. Dis..

[51] Kagiyama N., Mondillo S., Yoshida K., Mandoli G.E., Cameli M. (2020). Subtypes of Atrial Functional Mitral Regurgitation. JACC Cardiovasc. Imaging.

[52] Matta M., Ayoub C., Abou Hassan O.K., Layoun H., Cremer P.C., Hussein A., Schoenhagen P., Saliba W.I., Rodriguez L.L., Griffin B.P. (2021). Anatomic and Functional Determinants of Atrial Functional Mitral Regurgitation. Struct. Heart.

[53] Van De Heyning C.M., Magne J., Piérard L.A., Bruyère P.J., Davin L., De Maeyer C., Paelinck B.P., Vrints C.J., Lancellotti P. (2013). Assessment of left ventricular volumes and primary mitral regurgitation severity by 2D echocardiography and cardiovascular magnetic resonance. Cardiovasc. Ultrasound.

[54] Ünlü S., Duchenne J., Mirea O., Pagourelias E.D., Bézy S., Cvijic M., Beela A.S., Thomas J.D., Badano L.P., Voigt J.-U. (2019). Impact of apical foreshortening on deformation measurements: A report from the EACVI-ASE Strain Standardization Task Force. Eur. Heart J. Cardiovasc. Imaging.

[55] Schwammenthal E., Chen C., Benning F., Block M., Breithardt G., Levine R.A. (1994). Dynamics of mitral regurgitant flow and orifice area. Physiologic application of the proximal flow convergence method: Clinical data and experimental testing. Circulation.

[56] Sannino A., Fortuni F. (2024). Timing Surgical Mitral Valve Repair for Primary Mitral Regurgitation. J. Am. Coll. Cardiol..

[57] Fortuni F., Bax J.J., Delgado V. (2021). Changing the Paradigm in the Management of Valvular Heart Disease. Circulation.

[58] Tsang W., Lang R.M. (2013). Three-dimensional Echocardiography Is Essential for Intraoperative Assessment of Mitral Regurgitation. Circulation.

[59] Faletra F.F., Agricola E., Flachskampf F.A., Hahn R., Pepi M., Ajmone Marsan N., Wunderlich N., Elif Sade L., Donal E., Zamorano J.-L. (2023). Three-dimensional transoesophageal echocardiography: How to use and when to use—A clinical consensus statement from the European Association of Cardiovascular Imaging of the European Society of Cardiology. Eur. Heart J. Cardiovasc. Imaging.

[60] Levy F., Marechaux S., Iacuzio L., Schouver E.D., Castel A.L., Toledano M., Rusek S., Dor V., Tribouilloy C., Dreyfus G. (2018). Quantitative assessment of primary mitral regurgitation using left ventricular volumes obtained with new automated three-dimensional transthoracic echocardiographic software: A comparison with 3-Tesla cardiac magnetic resonance. Arch. Cardiovasc. Dis..

[61] Marechaux S., Le Goffic C., Ennezat P.V., Semichon M., Castel A.L., Delelis F., Lemahieu J.M., Menet A., Graux P., Tribouilloy C. (2014). Quantitative assessment of primary mitral regurgitation using left ventricular volumes: A three-dimensional transthoracic echocardiographic pilot study. Eur. Heart J. Cardiovasc. Imaging.

[62] Marsan N.A., Westenberg J.J.M., Ypenburg C., Delgado V., van Bommel R.J., Roes S.D., Nucifora G., van der Geest R.J., de Roos A., Reiber J.C. (2009). Quantification of Functional Mitral Regurgitation by Real-Time 3D Echocardiography. JACC Cardiovasc. Imaging.

[63] Piroli F., Boccellino A., Ingallina G., Rolando M., Melillo F., Ancona F., Stella S., Biondi F., Palmisano A., Esposito A. (2023). Feasibility and reliability of comprehensive three-dimensional transoesophageal echocardiography screening process for transcatheter mitral valve replacement. Eur. Heart J. Cardiovasc. Imaging.

[64] van Wijngaarden A.L., Mantegazza V., Hiemstra Y.L., Volpato V., van der Bijl P., Pepi M., Palmen M., Delgado V., Ajmone Marsan N., Tamborini G. (2022). Prognostic Impact of Extra–Mitral Valve Cardiac Involvement in Patients With Primary Mitral Regurgitation. JACC Cardiovasc. Imaging.

[65] Mentias A., Naji P., Gillinov A.M., Rodriguez L.L., Reed G., Mihaljevic T., Suri R.M., Sabik J.F., Svensson L.G., Grimm R.A. (2016). Strain Echocardiography and Functional Capacity in Asymptomatic Primary Mitral Regurgitation With Preserved Ejection Fraction. J. Am. Coll. Cardiol..

[66] Pio S.M., Medvedofsky D., Stassen J., Delgado V., Namazi F., Weissman N.J., Grayburn P., Kar S., Lim D.S., Zhou Z. (2023). Changes in Left Ventricular Global Longitudinal Strain in Patients With Heart Failure and Secondary Mitral Regurgitation: The COAPT Trial. J. Am. Heart Assoc..

[67] Mandoli G.E., Pastore M.C., Benfari G., Bisleri G., Maccherini M., Lisi G., Cameli P., Lisi M., Dokollari A., Carrucola C. (2021). Left atrial strain as a pre-operative prognostic marker for patients with severe mitral regurgitation. Int. J. Cardiol..

[68] Chang W., Wu N., Shih J., Hsu C., Chen Z., Cheng B. (2020). Right ventricular reserve post mitral valve repair is associated with heart failure hospitalization. Pulm. Circ..

[69] Hirasawa K., Izumo M., Akashi Y.J. (2023). Stress echocardiography in valvular heart disease. Front. Cardiovasc. Med..

[70] Lancellotti P., Magne J. (2013). Stress Echocardiography in Regurgitant Valve Disease. Circ. Cardiovasc. Imaging.

[71] Pierard L., Dulgheru R., Magne J., Lancellotti P. (2013). Dynamic ischaemic mitral regurgitation and the role of stress echocardiography. J. Cardiovasc. Echogr..

[72] Myerson S.G., d’Arcy J., Christiansen J.P., Dobson L.E., Mohiaddin R., Francis J.M., Prendergast B., Greenwood J.P., Karamitsos T.D., Neubauer S. (2016). Determination of Clinical Outcome in Mitral Regurgitation With Cardiovascular Magnetic Resonance Quantification. Circulation.

[73] Garg P., Pavon A.G., Penicka M., Uretsky S. (2025). Cardiovascular magnetic resonance imaging in mitral valve disease. Eur. Heart J..

[74] Ge Y., Gupta S., Fentanes E., Aghayev A., Steigner M., Sobieszczyk P., Kaneko T., Di Carli M.F., Bhatt D.L., Shah P. (2021). Role of Cardiac CT in Pre-Procedure Planning for Transcatheter Mitral Valve Replacement. JACC Cardiovasc. Imaging.

[75] Rolando M., Affronti A., Loreni F., Bergonzini M., Carluccio E., Fortuni F. (2025). Mitral Valve Repair in the Modern Era: Insights into Techniques and Technologies with a Glimpse of the Future. J. Clin. Med..

[76] Faletra F.F., Pasotti E., Moccetti T., Pedrazzini G. (2011). Real-time three-dimensional echocardiography during percutaneous edge-to-edge mitral valve repair. J. Cardiovasc. Echogr..

[77] Foster E., Wasserman H.S., Gray W., Homma S., Di Tullio M.R., Rodriguez L., Stewart W.J., Whitlow P., Block P., Martin R. (2007). Quantitative Assessment of Severity of Mitral Regurgitation by Serial Echocardiography in a Multicenter Clinical Trial of Percutaneous Mitral Valve Repair. Am. J. Cardiol..

[78] Fortuni F., Petrina S.M., Nicolosi G.L. (2025). Applicazioni dell’intelligenza artificiale nell’imaging cardiovascolare: Vantaggi, limiti e sfide future [Applications of artificial intelligence in cardiovascular imaging: Advantages, limitations, and future challenges]. G. Ital. Cardiol..

[79] Medvedofsky D., Mor-Avi V., Amzulescu M., Fernández-Golfín C., Hinojar R., Monaghan M.J., Otani K., Reiken J., Takeuchi M., Tsang W. (2018). Three-dimensional echocardiographic quantification of the left-heart chambers using an automated adaptive analytics algorithm: Multicentre validation study. Eur. Heart J. Cardiovasc. Imaging.

[80] Penso M., Pepi M., Mantegazza V., Cefalù C., Muratori M., Fusini L., Gripari P., Ghulam Ali S., Caiani E.G., Tamborini G. (2021). Machine Learning Prediction Models for Mitral Valve Repairability and Mitral Regurgitation Recurrence in Patients Undergoing Surgical Mitral Valve Repair. Bioengineering.

[81] Sacoransky E., Ke D.Y.J., Abuzeid W. (2024). Machine learning for prediction of transcatheter mitral valve repair outcomes: A systematic review. Inf. Med. Unlocked.

[82] Fortuni F., Ciliberti G., De Chiara B., Conte E., Franchin L., Musella F., Vitale E., Piroli F., Cangemi S., Cornara S. (2024). Advancements and applications of artificial intelligence in cardiovascular imaging: A comprehensive review. Eur. Heart J. Imaging Methods Pract..

[83] Hell M.M., Kreidel F., Geyer M., Ruf T.F., Tamm A.R., da Rocha e Silva J.G., Münzel T., von Bardeleben R.S. (2021). The Revolution in Heart Valve Therapy: Focus on Novel Imaging Techniques in Intra-Procedural Guidance. Struct. Heart.

[84] Fortuni F., Marques A.I., Bax J.J., Ajmone Marsan N., Delgado V. (2020). Echocardiography–computed tomography fusion imaging for guidance of transcatheter tricuspid valve annuloplasty. Eur. Heart J. Cardiovasc. Imaging.

[85] Melillo F., Fisicaro A., Stella S., Ancona F., Capogrosso C., Ingallina G., Maccagni D., Romano V., Ruggeri S., Godino C. (2021). Systematic Fluoroscopic-Echocardiographic Fusion Imaging Protocol for Transcatheter Edge-to-Edge Mitral Valve Repair Intraprocedural Monitoring. J. Am. Soc. Echocardiogr..

[86] Sadeghi A.H., el Mathari S., Abjigitova D., Maat A.P.W.M., Taverne Y.J.H.J., Bogers A.J.J.C., Mahtab E.A.F. (2022). Current and Future Applications of Virtual, Augmented, and Mixed Reality in Cardiothoracic Surgery. Ann. Thorac. Surg..

[87] Mao Y., Liu Y., Zhai M., Yang J. (2023). Application of and Prospects for 3-Dimensional Printing in Transcatheter Mitral Valve Interventions. Rev. Cardiovasc. Med..

[88] Zilio F., Giubilato S., Caldarola P., Ciliberti G., Di Monaco A., Dini C.S., Iannopollo G., Cornara S., Franchin L., Vitale E. (2025). The role of simulation in medical education, clinical risk management, and enhancing patient care in cardiology. G. Ital. Cardiol..

